# Access to primary and secondary health care services for people living with diabetes and lower-limb amputation during the COVID-19 pandemic in Lebanon: a qualitative study

**DOI:** 10.1186/s12913-022-07921-7

**Published:** 2022-05-03

**Authors:** Lea Chaiban, Aicha Benyaich, Sally Yaacoub, Haya Rawi, Claudia Truppa, Marco Bardus

**Affiliations:** 1 American University of Beirut, Beirut, Lebanon; 2 International Committee of the Red Cross (ICRC), Jeanne D’Arc 326 Building, Sidani Street, Hamra, Beirut, Lebanon; 3 CRIMEDIM - Center for Research and Training in Disaster Medicine, Humanitarian Aid and Global Health, Novara, Italy; 4 School of Health, Sport, and Life Sciences, Leeds Trinity University, Horsforth, Leeds, UK; 5 Department of Health Promotion and Community Health, American University of Beirut, Beirut, Lebanon

**Keywords:** Disability, Lebanon, Diabetes, Lower-limb amputation, Access to healthcare, Pandemic

## Abstract

**Background:**

People living with chronic conditions and physical disabilities face many challenges accessing healthcare services. In Lebanon, in 2020, the COVID-19 pandemic and concomitant economic crisis further exacerbated the living conditions of this segment of the population. This study explored the barriers to accessing healthcare services among people living with diabetes and lower-limb amputation during the pandemic.

**Methods:**

We conducted semi-structured, in-depth phone interviews with users of the Physical Rehabilitation Program, offered by the International Committee of the Red Cross. We used a purposive sampling technique to achieve maximum variation. Interviews were audio-recorded, transcribed, translated, and analyzed using thematic analysis following the “codebook” approach. Transcripts were coded and grouped in a matrix that allowed the development of themes and sub-themes inductively and deductively generated.

**Results:**

Eight participants (7 males, 1 female) agreed to be interviewed and participated in the study between March and April, 2021. Barriers to healthcare services access were grouped according to five emerging themes: (1) economic barriers, included increasing costs of food, health services and medications, transportation, shortage of medications, and limited income; (2) structural barriers: availability of transportation, physical environment, and service quality and availability; (3) cultural barriers: marginalization due to their physical disabilities; favoritism in service provision; (4) personal barriers: lack of psychosocial support and limited knowledge about services; (5) COVID-19 barriers: fear of getting sick when visiting healthcare facilities, and heightened social isolation due to lockdowns and physical distancing.

**Conclusion:**

The underlying economic crisis has worsened the conditions of people living with diabetes and lower-limb amputation. The pandemic has made these individuals more vulnerable to external and contextual factors that cannot be addressed only at an individual level. In the absence of a protective legal framework to mitigate inequalities, we provide recommendations for governments and nongovernmental institutions to develop solutions for more equitable access to healthcare for this segment of the population.

**Supplementary Information:**

The online version contains supplementary material available at 10.1186/s12913-022-07921-7.

## Background

In 2020, more than 1 billion people were living with a disability, which accounts for an estimated 15% of the global population [1]. With the rise in chronic and other illnesses, this number is expected to increase over time [2]. On average, people with disabilities have poorer health, less access to education, lower work opportunities, and are at higher risk to live in poverty compared to people with no disabilities [2].

In Lebanon, 10–15% of the Lebanese population lives with either physical, sensory, intellectual, or mental disabilities [3]. The number of persons with disabilities (PWDs) has increased with the influx of refugees in the country. According to the most recent Vulnerability Assessment for Syrian Refugees (VASyR) report in Lebanon, 33% of the 4563 interviewed households reported having at least one household member with a disability [4]. An earlier study showed that among PWDs, 14 and 20% of Lebanese and Syrian respondents, respectively, suffer from non-communicable diseases such as diabetes, hypertension, cardiovascular diseases, and asthma [5]. Diabetes is considered a major chronic illness, affecting about 13% of the local adult population [6], and around 10% of Syrian refugees [7]. Besides worrying about glycemic levels, healthy eating, and medical appointments, people with diabetes can develop neuropathy and poor circulation which can result in foot ulcers. This can further be complicated by skin and soft tissue infections that can evolve into osteomyelitis [8]. Untreated or mismanaged foot ulcers can lead to tissue necrosis, which requires foot amputation. Consequently, the person would suffer from a physical disability along with their diabetic condition. In fact, in Lebanon, around 59% of surgical amputations are due to diabetes-related complications [9].

People with physical disabilities are among the most vulnerable groups and are at a higher risk of being excluded from healthcare services [10]. Accessing healthcare services is a human right. The Convention on the Rights of Persons with Disabilities (CRPD) states in Article 25 that “people with disabilities have the right to the enjoyment of the highest attainable standard of health without discrimination on the basis of disabilities” [11]. In Lebanon, Syrians and Lebanese with disabilities have access to primary health care (PHC) services through the Ministry of Health, and Palestinians receive their services through the United Nations Relief and Works Agency (UNRWA). The Ministry of Social Affairs (MoSA) also issued disability cards for Lebanese citizens only who live with disabilities as per law 220/2000 on the Rights of Disabled Person. According to MoSA, the cardholders are “entitled to a wide range of healthcare services, including primary, secondary and rehabilitation services, to be covered in full by the relevant ministries”, however, this coverage does not include tertiary level of care. The enforcement of the provisions of this law has been partial and slow-paced [12]. The severe financial crisis affecting the country has further hindered the public system’s capacity to guarantee a timely provision of reimbursement of services to the different providers. The uncertainties related to the timeframe of reimbursement have made it more frequent for some providers to deny requests from disability cardholders, or to request anticipated payments for service provision [13].

In 2015, the International Committee of the Red Cross (ICRC) in Lebanon established a Physical Rehabilitation Program (PRP) that offers mobility assistive devices (e.g., prostheses, orthoses, wheelchairs) and physiotherapy services. ICRC mainly supports the population who are not entitled to receive services through the local health authorities, namely refugees and vulnerable local communities. In 2019, ICRC assisted 868 people across Lebanon and provided more than 1180 assistive devices [14].

The literature highlights how COVID-19 made it more challenging for patients to receive adequate diabetic foot care from a healthcare system that has been focused on mitigating the spread of the virus [15]. The lockdown showed to interrupt diabetic foot care and lower limb preservation. France, for example, has reported how the pandemic created a barrier for patients with diabetic foot ulcers in receiving care after a marked drop in hospitalization rate among diabetic foot ulcer patients [16]. Besides delayed diagnosis, the global literature, has also reported how the pandemic made it even more difficult for people with disabilities to access health services creating multiple layers of complexity and inter-related barriers [17, 18]. A study done in Southern India reports how PWDs living with diabetes could not visit their physician during the lockdown and had to use old prescriptions for their medication [19]. In Bangladesh, PWDs inclusiveness was raised when COVID-19 testing was not easily accessible and the country experienced a wide shutdown of rehabilitation essential services, excluding PWDs from the pandemic’s preparedness, response and mitigation plan [20]. The existing literature about people living with disabilities in Lebanon before the pandemic showed that they faced many barriers to accessing healthcare services, including financial ability, limited information about certain services, structural and cognitive barriers [3, 5, 10]. However, since 2019, the country has been in a looming economic crisis which is further exacerbated by the Beirut explosion, the COVID-19 pandemic, and the consequential lockdowns [21]. To the best of our knowledge, no study has yet attempted to describe how the pandemic and the economic downturn have affected the lives of people living with chronic conditions and physical disabilities.

This study aims to provide an in-depth understanding of the compounding effects of the different colliding crises in Lebanon on access to health care services for people living with chronic conditions and physical disabilities. In particular, it aims to describe the different challenges faced by people living with diabetes and lower-limb amputation in accessing primary and secondary healthcare services during the COVID-19 pandemic in Lebanon.

## Methodology

We adopted a qualitative research design to garner an insight into the different perspectives, experiences, and attitudes of the participants [22]. We conducted in-depth semi-structured interviews and the generated transcripts were then analyzed using thematic analysis (TA) following the “codebook” approach [23, 24]. This TA approach allows for flexibility where both inductive and deductive approaches are used to analyze the qualitative data which helped us understand the participants’ situation and to have a sense on their living experiences and challenges [24].

### Recruitment

We sought to recruit people who received physical rehabilitation services from the ICRC in Lebanon between January 2015 and December 2019. The target population included all persons above 18, with lower-limb amputation and diabetes. There were 56 individuals in total (15 females and 41 males) who fulfilled these main inclusion criteria. Thirty-five were classified as adults (18 and above) and 21 were older adults (65 and above). PRP participants were of different nationalities: Lebanese (42%), Syrian (38%), and Palestinians (20%). Geographically, they were distributed as follows: Beirut/Mount Lebanon (BML) (18%), Beqaa (27%), North (27%), and South (28%). We used a purposive sampling technique to achieve maximum variation in the sample of interviewed participants. We aimed to interview at least one person from each governorate and of each nationality. The ICRC team invited the participants to take part in this study in March 2021 following a standardized invitation script, and the team stopped recruiting when at least three participants per governorate agreed to participate.

### Data collection

In-depth semi-structured interviews were conducted during March and April 2021 within 3 weeks after the study’s invitation. To comply with COVID-19 guidelines, the co-investigator (LC) conducted 30 to 55-min semi-structured phone interviews in colloquial Arabic following a structured, predefined interview guide (Additional file 1). The interviews were digitally recorded after obtaining the participants’ oral consent and then deleted after transcription. The questions covered topics related to the healthcare services that participants seek related to their disabilities and chronic condition. The topics also covered the challenges people face in receiving treatment of care during the COVID-19 pandemic.

### Data analysis

The in-depth interviews were audio-recorded, translated, and transcribed into English. The transcripts were analyzed using a “codebook” thematic analysis [24]. Thematic analysis interprets data by identifying patterns to answer a research question. “Codebook” TA uses a somewhat structured coding framework to develop the analyzed data. Themes are usually generated early on and then refined by new themes that are inductively developed from the data. Following Braun and Clarke’s guide, “Codebook” TA was conducted by the first author (LC) where she first immersed herself in the data by reading every transcript. The transcripts were coded and then grouped into categories in a matrix, following the overarching themes (deductively generated). The primary investigator and sixth author (MB) and the first author (LC) both discussed and agreed on the generated themes and sub-themes, which were mainly deducted from the interview guide, and other themes were generated inductively from recurring patterns. The whole team then met to review and validate the developed themes and subthemes.

### Ethical considerations

This study was implemented after being reviewed and approved by the Institutional Review Board (IRB) at the American University of Beirut (SBS-2020-0532). ICRC personnel following an invitation script invited participants and shared contact details of those interested with the first author. All candidates had equal chances to participate in the interviews as they were randomly invited to participate. The first author contacted interested participants and obtained their oral consent to be interviewed. Participants were assured that all the data collected is confidential and accessible to the research team. The interviews were digitally recorded, securely saved on a private password-protected computer, and then deleted once transcribed. The oral consent script was shared with the participants via WhatsApp, including the researchers’ numbers and Embrace’s hotline number for free mental health support when needed.

## Results

### Participant characteristics

Out of the 56 eligible persons, the ICRC team reached out to 25 persons. Of these, 14 agreed to be contacted for an interview. When the interviews took place, out of the 14 persons, one person passed away, two were unreachable, one was no longer interested, and 10 gave their oral consent. Out of the 10 participants who agreed to be interviewed, eight completed the full interview and two participants withdrew their consent during the dialogue and therefore did not complete it. Table 1 reported the characteristics of the interviewed individuals. Most of the participants were Lebanese males and resided in Beirut/Mount Lebanon (BML). Only one female participant completed the interview through her male caregiver. No Palestinian participants completed the interview as they were not available after initial contact.

**Table 1:** Characteristics of informants

Variables	Accepted the Invitation (***n*** = 14)	Completed the Interview (***n*** = 8)
**Sex**		
***Female***	3	1^a^
***Male***	11	7
**Residence (Governorate)**		
***Beirut/Mount Lebanon***	6	4
***North***	3	2
***South***	2	1
***Beqaa***	3	1
**Nationality**		
***Lebanese***	8	5
***Syrians***	3	3
***Palestinians***	3	0

^a^Male caregiver was the dominant speaker in the interview

### Barriers to health care services for participants

**Fig. 1:**
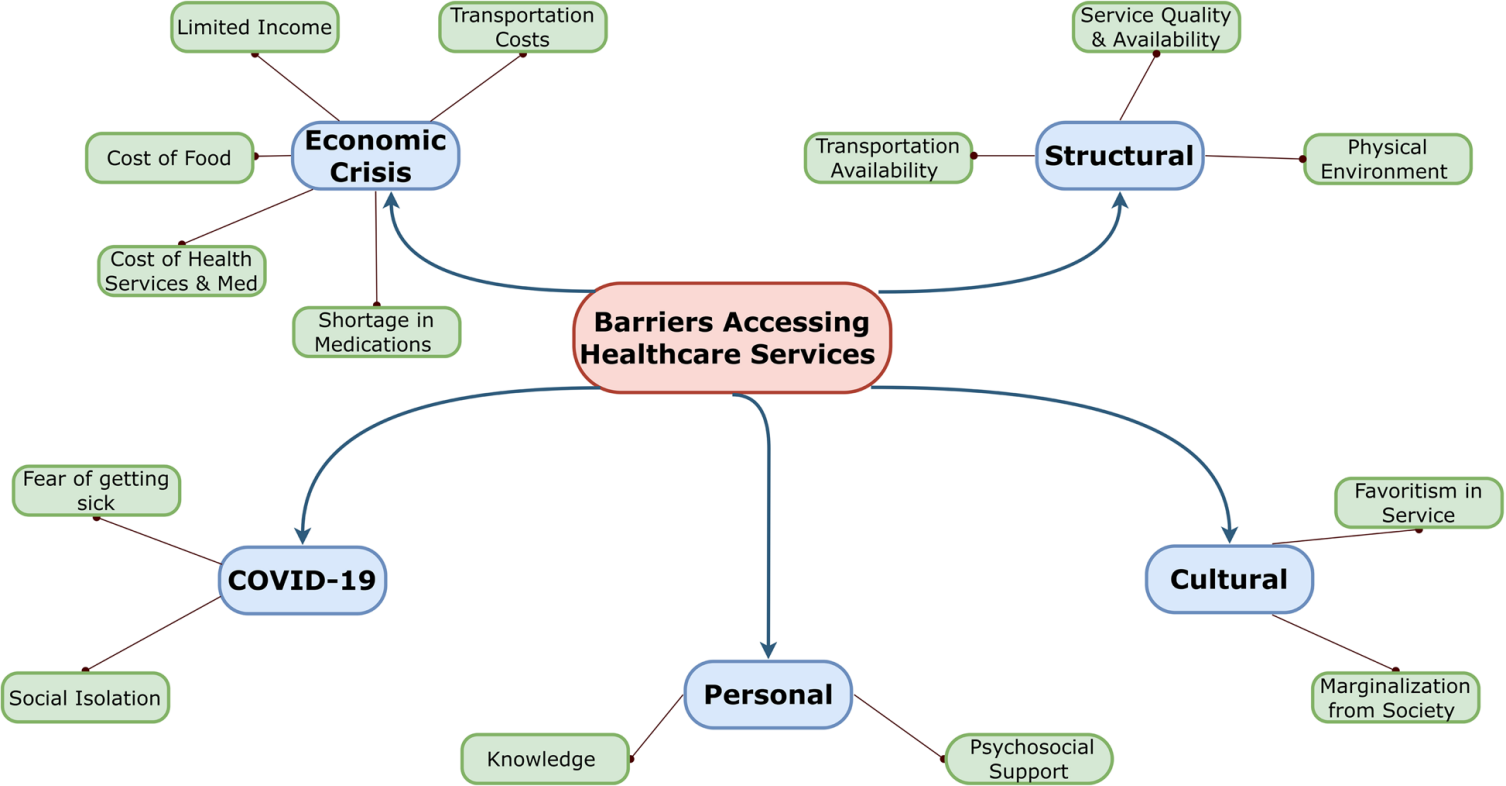
Mind map of identified barriers to health care services for participants

The barriers to accessing health care services for the people enrolled in the ICRC program were arranged under five overarching themes: economic crisis, structural, cultural, personal, and COVID-19 related barriers (Fig. 1). Each theme and sub-theme will be described in the following paragraphs, providing exemplar quotations, where applicable.

## Economic crisis

### Limited income

All participants reported that they did not have a job or had lost their job due to their disabilities. Participants were mainly reliant on the income of the caregivers or financial aid received from organizations or friends. Due to the current economic collapse and the fluctuating dollar exchange rate, respondents expressed that their financial income had become so limited that insuring food for the day had become their priority for the time being.

“[Aid organizations] would give me 300,000 LBP which was 200$ and that used to get me a lot, but now a 300,000 LBP is 30$, which can’t get you a bottle of oil anymore… if diabetes is an illness, then sadness is an illness too” (Participant #7)

“I used to be a taxi driver, but now I am not and with the current situation I can’t get my medications, I am not even able to pay rent, the landlord will kick me out soon… everything is on top of everything” (Participant #1)

### Cost of food

Healthy eating is key for managing diabetes and participants expressed the importance of taking care of their diet by cutting down on excessive sugar and carbohydrates; however, they explained that their current financial situation obliged them to opt for food of lower quality and less diversity, making it even more difficult to regulate their sugar levels.

“The problem is that I don’t have an income, I don’t work, and I don’t have anyone who can cook for me. I try to cook stuff like beans, rice, fried potato… And all these have carbs and they are not good for me… It has been more than a year I haven’t eaten chicken or meat. I once craved a chicken breast, I called the market and told me it costs 38,000LBP… I told him to keep it for themselves.” (Participant #4)

### Cost of health services and medications

None of the participants had private health insurance and only one person benefited from the National Social Security Fund (NSSF). Hospital expenses were usually covered by the patients themselves. The participants relied on some organizations that partially or fully covered medical expenses. In addition, some reported receiving financial support from friends or relatives. For most refugees, humanitarian aid agencies cover medical expenses. For example, some participants declared that they have not visited their physician in a while and still follow their original/initial prescription due to their inability to pay for a consultation.

“I don’t have insurance nor NSSF… I was once not feeling well, my friend offered me a ride to the hospital, but I couldn’t [afford expenses] so I went to sleep, and I just cried for God’s help” (Participant #1)

“I stayed 8 months in the hospital, my fee was 14 million 900 thousand, my friends started a campaign, and asked for donations from people to collect the needed money. The hospital would not discharge me until I pay the remaining. My daughter’s godfather then paid the rest… I am still in debt to that person. How am I going to pay him? (Participant #4)

Not all PHCs offered services that include medical imaging and blood exams. In most cases, participants had to pay for these services out of pocket. Persons with diabetes require constant medical tests to monitor their blood glucose level and other conditions related to diabetes which have become more expensive. Participants living in Beirut also reported that medical costs in the city were much higher than hospital services outside the city. For that reason, their caregivers were willing to drive them an extra hour just to get services at a lower cost. On the other hand, physical therapy and prosthetics were essential services for PWDs to regain their mobility and interviewees mentioned that ICRC covered all expenses related to these services, yet other challenges mentioned below limited their access to these services.

“Here in Beirut, [hospital service] is expensive, and us, God help us, as they say, follow the cheap. Chtoura (in Bekaa) is cheaper.” (Participant #6)

“It has been more than 8 months since I have not done any medical tests. Because how am I going to afford that. I don’t have the money.” (Participant #4)

Regarding medications, most of the interviewees reported that the prices of medications had slightly increased. Some of the interviewees got their medication from PHCs or organizations at a reduced price or sometimes for free, but not all medications were available.

“My meds are expensive, and usually expensive meds are not found at PHCs” (Participant #8)

### Transportation costs

Many people with physical disabilities reported that one of the major barriers in accessing healthcare services was the expensive transportation cost. As mentioned above, several expressed that some associations and organizations offered free, or partially free health services; however, the transportation cost remained a challenge to be considered. When participants had no one around to give them free rides, they had to pay for public transportation such as *service* (shared taxis), call a private taxi, or even hire a driver. Even though some expressed that organizations helped them cover transportation fees, it was not the case for everyone. For that reason, some of the interviewees reported falling short in following up with their physiotherapy sessions for example.

“To get to the center, I need 3 to 4 *service*… In Tripoli, the fare for a *service* ride was known to be cheaper than other places, but now they are pricing it 4,000LBP and 5,000LBP... I understand. The situation is so bad.” (Participant #1)

“You are opening back deep wounds (*laughs*), yes I used to pay for each round trip to the [physio] center 75,000 LBP. This was before the dollar crisis… at some point, I used to go daily, but now I am having my physio sessions at home.” (Participant #8)

“My physio session could cost 30,000 LBP but my car ride would cost 30,000 LBP so, I try to do some [exercises] home by myself, what can I do.” (Participant #2)

### Shortage in medications

Almost all participants mentioned that some of their medications were no longer available or are harder to find. They sometimes had to search several pharmacies to find their medications. Others sought PHCs and organizations to find generic medications and some were left with no medication. A lot of the participants and caregivers were anxious about running out of prescription drugs, therefore they always tried to get more than they needed, but not everyone had the means to do that.

“There is indeed a shortage in medications, but if someone has the means to go around and search well, one can find them.” (Participant #1)

“They [PHC] only give me two insulin shots though each pack has 4 syringes. So, I go to other PHCs with my prescription, and I keep searching until I find one more syringe so I can secure my need” (Participant #3)

“...it [drug] used to be available in pharmacies, but now you need to put your name and every month you are only allowed to get one pack, the situation has become a bit hard.” (Participant #6)

## Structural barriers

### Transportation availability

Not all the interviewees owned a car, and if they did, none of them were able to drive their cars because of their disability. Some health institutions were said to be near and other participants expressed the long distance they had to travel to get to their destination. In both cases, the participants’ disabilities obliged them to be dependent on others for a ride or to seek other modes of transportation such as private taxis, shared taxis, and sometimes ambulances. Some participants reported receiving certain services at home and would not worry about transportation.

“The PHC is 1 km or more away, I need a car to give me a ride. I go out on the streets, and I pray for God to send me a car. Someone who is passing from one village to another asks me where I am going and offers the ride. Sometimes people offer to wait for me so they can give me a ride back. I once even got with someone on his motorbike” (Participant #3)

“The center is only 10-15 min away from my home, but I can’t drive yet, my right leg is amputated from above my knee, so my nephew is giving me a ride now” (Participant #5)

“Back then I had a Taxi… I drove it and passengers never knew I was amputated, but I had to get rid of the car, and I have no one to give me rides to the physician” (Participant #1)

The issue of transportation was one of the main reasons why people with disabilities would miss their clinic appointment because they were highly dependent on the ride’s availability. Caregivers had to find the time to give them a ride, some had to skip work to be able to do that.

“Sometimes my neighbor promises me a ride, but he doesn’t come, so I have to wait the next day to make another appointment” (Participant #3)

“The appointments were from 10-12, so yes of course I [caregiver] had to leave my job so I can go with her [PWD]. There is no one but me, what shall I do, who is going to take her” (Participant #6)

### Physical environment

All participants agreed that hospitals and other health institutions were sufficiently accessible for people with physical disabilities. They were equipped with ramps and elevators and health care providers assisted people with wheelchairs when needed. However, one of the main issues highlighted by one participant was that institutions, especially in Beirut, did not have nearby parking spots for him to get out of the car at his own pace, although a few provided assigned parking spaces for people in wheelchairs.

“I was doing my physio sessions at that clinic. We couldn’t find a place to park, so we stopped the car at the side and the cars behind us had to wait for me to step out. My sister then waited for me in the car beside the clinic and told the security about my disability and that there was no place to park, but he yelled at her and told her that she is causing a lot of traffic. No respect.” (Participant #5).

Several interviewees explained that their main physical barrier was at their place of residency. The presence of stairs seemed to be an important barrier for them to go out, and for that reason, they needed to call someone such as neighbors, friends, or even paramedics to carry them down to the street.

“I have some difficult stairs here at my doorstep. It is challenging to go out, so I need someone to carry me out. I call sometimes the Red Cross; they carry me, and they give me a ride when needed, but I don’t prefer having people carrying me down the stairs, so I prefer getting services at home.” (Participant #8)

“My issue is to go out of the house as I have two steps at my front door because I still can’t go up these stairs easily… If there aren’t some men outside my street who could carry me, I can’t just go out on my own...and when I get back (from the clinic) I have to wait in the streets for two of my friends to carry me back home” (Participant #4)

### Service quality and availability

Almost all the respondents praised their current physicians. They believed that their doctor was caring, supportive, and was always ready to answer their questions. Participants perceived health workers to treat PWDs with compassion only because it was part of their job and were somehow obliged to. Nevertheless, many interviewees who shared their amputation experience perceived inappropriateness of the treatment plan. They were wondering whether their leg could have been saved had the possibility of accessing a different hospital at a different time. One participant living away from the city also reported that nearby hospitals were not equipped with the services he needed such as specialized medical imaging, so he had to travel farther to more expensive hospitals in the cities to get the care needed.

“Thank God *(sarcastically)*, our hospitals in the North are under-resourced. I had three operations, and no one figured I had diabetes. The doctor first did my operation by mistake. Once I checked with another Dr. and he told me that I didn’t need it [amputation]. My situation could have been resolved by medications within days” (Participant #1)

“My leg was hurting me a lot; I spent a whole month at this public hospital, and they didn’t do anything. The doctor kept telling me that antibiotics will solve the problem… I then went to a private hospital for my open-heart surgery and the doctor saw me limping. He asked me to remove my socks and there was a black dot on my leg. He put his hand on his forehead and said: ‘son you came in late’” (Participant #4)

Half of the participants mentioned that their prostheses and/or their walking device were uncomfortable, therefore they were not wearing them much. Persons with diabetes explained that due to their amputations, they are more sensitive to bruising at the stump level which takes a longer time to heal. This makes it more challenging to walk independently for a longer period and even limits their movements inside the house.

“Because I have diabetes, I am not able to wear my device properly. Each time I wear it, it gives me bruises. If I didn’t have these bruises, I would have walked and gotten out” (Participant #8)

“I wore it [prosthesis] two-three times at home but it was not comfortable at all. I used to wear it for some time and go out and visit friends, but now I am staying home” Participant #4)

The participants who were refugees struggled with additional challenges that were not mentioned by Lebanese citizens. They reported that, sometimes, hospitals refused to admit refugees, so they had to seek other hospitals where humanitarian agencies would cover their expenses.

“I got corona (COVID-19) 4 months ago. My friend took me to the hospital, but the hospital doesn’t accept Syrians, so I had to go to another hospital. They wouldn’t allow me to enter the emergency room before I pay a certain amount, so I had to wait for the UN’s approval to get my expenses covered.” (Participant #2)

## Cultural barriers

### Marginalization from society

Participants described how society marginalizes and stigmatizes PWDs. The public’s attitude and downward look towards PWDs pushed them even more outside the community, enabling vulnerable groups to fully participate in society. Respondents explained how they always felt disregarded and excluded in terms of access to services and opportunities, especially in employment. Interviewees discussed the insufficient interventions done by associations to meet the social needs of PWDs, highlighting that health care does not end at hospital discharge. According to them, more programs and opportunities were needed to reintegrate them back into society.

“I tried to look for work opportunities everywhere, but I failed because I use a walker, and no one would employ me… people who have disabilities can work and can give back, but they don’t have opportunities” (Participant #2)

“Society looks at you with pity. They say, ‘look where he was and look where he is now… poor him he doesn’t have anyone around’ and this pettiness comes to no use.” (Participant #1)

“People assume I will be depressed and expect me to hide my leg… but I am doing well, and life is normal, this is normal and there is nothing to be ashamed of.” (Participant #5)

### Unfairness in treatment / favoritism in service provision

Respondents who received care from PHCs or aid from organizations reported witnessing unfairness in service delivery; from accessing medications, getting a doctor’s appointment, or even receiving financial aid. They repeatedly mentioned being excluded and getting empty promises.

“At the PHC, I usually wait a lot for my turn because whoever has ‘wasta^1^’ will go in first” (Participant #3)

“Each time I put my name to receive help, I never get a reply back. I feel like I am always excluded, thank God” (Participant #1)

## Personal barriers

### Need for psychosocial support

All the interviewees preferred to have someone by their side for physical or emotional support and not having company was a valid reason for them not to seek health care services. For some, having a companion gave them the physical support they needed, such as assistance in balance and stability. For others, they expressed that having a trusted person around for emotional support was enough for them to be comfortable. While participants preferred to have someone by their side, they also expressed feeling as if they were an extra burden on their caregivers. One participant pointed out that his family cannot serve him forever and recommended having special services like companions, drivers, or a car. Even the ones who did not have family around found it harder to ask for help, especially when everyone is being affected by the country’s dire economic crisis. Most of the interviewees agreed that psychological support was important for people who needed it, especially in the early phases of their amputation as one could suffer from depression and suicidal thoughts. However, no one perceived that this kind of support is very valuable today. Participants claimed to have strong faith and are usually joyful and believed that working and independently ensuring their daily bread would automatically improve their mental health, without the help of a specialist.

“I would prefer to have someone beside me to support me emotionally, and if something happens to me, God forbids, I have someone next to me who I trust. I am the type of person who is shy to ask from people I don’t know.” (Participant #1)

“I am diabetic, and my sugar level might fluctuate. I must always have company because when my sugar level drops, I fall and if I fall, I can hurt myself” (Participant #3)

“We need to be independent, no matter how close you are with your mother or siblings at some point they will get tired of you… I sometimes want to go out, but I am not going to oblige my brother to take me and drop me off each time” (Participant #5)

“*(sighs)* mental health support could be useful for youngsters who are 15-30 years but a person my age, what would mental support do for me… I know what is wrong with me, I wouldn’t want someone explaining this to me again. It is going be more painful...I already know what my mental health is like. The issue is not affording basic things like food.” (Participant #7)

### Knowledge about condition and services

All the interviewees showed a basic understanding of diabetes and how to control their blood sugar. In case they needed more health information, most of the participants would call their doctors and a few of them mentioned that they referred to organizations. Still, some respondents found it challenging to access information regarding the services offered by the government and other non-profit organizations to PWDs. They were not sure who to ask or where to go.

“I was wondering if the ministry of social affairs would help me, and someone promised me to call me back and help me to put my name for aid… I need someone to guide me regarding the services” (Participant #5)

## COVID-19- related barriers

### Fear of getting sick

Most participants exhibited anxiety regarding going out because, as people with diabetes, they were at a higher risk of developing severe disease following COVID-19 infection. For some, COVID-19 did not stop them from reaching health care services while taking the right precautionary measures each time they needed to make it to a health institution. Nonetheless, some expressed the fear of leaving their homes and getting infected. They were trying to limit all health care services to those that can be provided at home.

“I am not going out because here... I am scared. I am so scared. I am scared not only of getting corona but of falling and needing hospitalization and then getting corona from the hospital” (Participant #4)

“Once, I was in the *service* and this woman kept saying that she had corona, and she was sitting beside me. The whole ride I was so scared… I am now terrified if I got it” (Participant #2)

### Social isolation

The lockdown measures and the need to reduce physical contact limited the participants’ mobility, making them more isolated and lonelier, especially with restrictions on home visits. Participants were walking less, thus making their healthy leg weaker and in need of more physiotherapy sessions.

“This bothers me. No more visits. If someone wants to check up on me, they should call me. Before corona, my house was never empty. Now, I stay home alone all by myself, my wife works and comes home at 5:30” (Participant #8)

“Because of the corona (COVID-19), I am home all day, and no one visits. It has been more than a month and a half that I haven’t worn [the prosthesis]” (Participant #4)

## Discussion

The results of the eight in-depth semi-structured interviews conducted in this study illustrated a complex web of challenges affecting the access to primary and secondary healthcare services during the COVID-19 pandemic among people living with both diabetes and lower limb amputation in Lebanon.

## Summary and interpretation of findings

### Participants’ characteristics

Almost all participants were male; even though the literature reports a significant association between male gender and an increased risk of amputation compared to female persons with diabetes [25], the WHO reports that disability is slightly more common in women than in men (19.2% of women and 12% of men) [26]. Globally, women make up three-fourths of persons with disabilities in low and middle-income countries (LMIC) [27]. However, in Lebanon, out of the 114,000 persons with disabilities (holding a disability card from MoSA), 62% are males and only 38% are females [28]. Consequently, there is a discordance between global-level data and local-level data. The gender distribution of our included participants might be related to the cultural norms and social traditions women with disabilities are affected by in a developing country such as Lebanon. Women with disabilities are recognized to be multiply disadvantaged, experiencing exclusion on account of their gender and their disability [29]. They are more prone to abuse, maltreatment, and negligent treatment [30].

As we were not able to capture the women’s perspective on access to health services, we believe that it would have been concordant with the global literature that women have lower access to health care. In general, women have different health needs compared to men [31]. They have a higher risk of violence, lower access to health care (primary, secondary, and sexual reproductive health care), and exposure to risks related to water and sanitation. “Women with disabilities, of all ages, often have difficulty with physical access to health services. Women with mental disabilities are particularly vulnerable, while there is limited understanding, in general, of the broad range of risks to mental health to which women are disproportionately susceptible as a result of gender discrimination, violence, poverty, armed conflict, dislocation, and other forms of social deprivation” [32].

### Economic crisis

One of the main barriers discussed by all the interviewees was Lebanon’s current economic crisis. Lebanon has rushed into a financial crisis since the end of 2019 where a fast cut in capital inflows triggered the collapse of the banking sectors and the exchange rate [21]. In early 2021, the Lebanese lira has lost more than 80% of its value resulting in major price increases across goods and services [33]. The economic crisis has been further exacerbated by the COVID-19 pandemic and the August 4, 2020, Beirut Port blast, pushing more than half of the population below the poverty line and resulting in many households struggling with access to basic services, besides healthcare [21]. In 2020, the vulnerability of Syrian refugees had also worsened after 90% of the households were found to live under the extreme poverty line compared to 55% in the previous year [4]. Participants reported that they did not have a sufficient income, and many were financially dependent on their caregivers or friends and relatives, knowing that only 20% of PWDs were employed in 2016, 5 years before the current financial crisis [34]. The increase in prices of basic foods made it difficult for people with diabetes to maintain a healthy and diverse diet, low in carbohydrates and sugar [35]. Besides maintaining a balanced diet, people living with diabetes and physical disabilities needed access to several health services, including medication, medical consultation, physical therapy sessions, blood tests, medical imaging exams, and hospitalization in case of development of complications. Respondents noted a shortage in medications and an increase in anxiety that came with struggling to ensure their monthly needs. With the devaluation of the Lebanese Lira, pharmaceutical importers have been falling short in supplying drugs since June 2020 and patients have been substituting their medications with generic ones when possible and available [36].

The financial barrier was a major obstacle in accessing healthcare among PWDs. This is consistent with the results from a literature review on barriers to healthcare services for people with disabilities in developing countries, which also highlighted the cost associated with getting to and receiving healthcare as one of the main obstacles [10]. A policy brief on improving healthcare access for persons with disabilities in Lebanon reported that the most commonly cited barrier to accessing healthcare among respondents was financial ability (78.5%) [12]. In fact, in our study, interviewees reported having neither insurance nor NSSF as a vital reason for not being able to access healthcare services. They elaborated on the need to pay for these services out of pocket or go to some services that are partially or fully covered by humanitarian organizations or PHCs whenever available. The Lebanese Social Security system has “serious shortcomings in terms of coverage” where around 40% of the Lebanese population is outside any insurance system, 40 to 50% are not registered in the National Social Security Fund, and only 8% have private insurance [37]. The decree of Law 220/2000 has not been issued leaving the most vulnerable groups, like PWDs, outside the framework of the coverage [37]. As for refugees with disabilities, humanitarian agencies like the United Nations High Commissioner for Refugees (UNHCR) partially subsidize some health services, and patients must cover the rest [12]. Even though some stated to receive free services from PHCs and other organizations, transportation was still an important obstacle brought up that is consistent with other findings in Lebanon [5, 10] and developing countries [38]. Many participants noted the increase in the cost of transportation in both public and private ones. Healthcare institutions were not possible for PWDs to be accessed through walking. No one drove on his or her own, so they sometimes missed their appointments as their ride was dependent on either the money available in their pockets or on someone else’s schedule.

### Structural barriers

Contrary to other studies conducted in Lebanon, none of the interviewees reported that local healthcare centers lacked proper infrastructure, such as elevators and ramps to accommodate them [5]. They all agreed that facilities were well equipped to a certain extent. One respondent, however, raised the issue of not finding a tight parking spot. Law 220/2000 considers it a right to have close and accessible parking spots for PWDs, yet centers in congested areas lack this right [39].

However, the study found a recurring barrier at the household level. Participants raised the concern of not being able to overcome their front doorsteps, which for some was a barrier for leaving their homes by themselves in the first place. Amputees had to always rely on friends, family, neighbors, or even paramedics to carry them down the stairs, an undesired feeling expressed by everyone.

Participants were generally positive regarding the attitudes of healthcare providers, predominantly with their physicians, but some acclaimed that their amputation was a consequence of health providers’ malpractice. Participants expressed that some hospitals, especially rural and public ones were not financially, nor resources supported to deliver high-quality care [40]. PWD sometimes had to travel farther to receive the care they needed because health facilities in rural regions, as also mentioned by the interviewees, were under-equipped and sometimes lacked the required service [41]. Refugees also pointed out that certain hospitals refused their admission, limiting their access to certain institutions even when it is their right to access any. Health facilities and hospitals are overburdened, preventing them from offering adequate services to citizens and refugees [42].

Rehabilitation is crucial to meet the needs of PWDs and gain independence, yet rehabilitation among amputees with diabetes is challenging. Since one of the comorbidities related to diabetes is the delay in wound healing, sores and infections common in the stump can create an interruption in the rehabilitation process [43].

### Cultural barriers

Stigmatization and marginalization were considered essential barriers in accessing healthcare services aligned with studies conducted in developing countries [10, 38]. The Lebanese society perceives PWDs as passive community members; conversely, PWDs feel isolated and struggle to find opportunities to be active participants and give back to the community [44]. The community’s attitude towards PWD amplifies the feelings of shyness, rejection, and lack of confidence, and these negative feelings drive PWDs to become ashamed and not leave their houses to obtain proper health services [10, 45]. Favoritism or ‘wasta’ in Arabic was an additional issue raised in this study where interviewees expressed how they witnessed inequitable service delivery in some PHC and organizations. Favoritism echoes with previous findings not only in accessing services but also in granting and distributing a disability card [3, 5].

### Personal barriers

The lack of assistance to accompany a PWDs was another barrier that resonated with findings reported in the literature [38, 46, 47]. A meta-synthesis of qualitative studies exploring the barriers to primary healthcare services experienced by PWDs in LMICs reported that PWDs often feel the need to be accompanied by family members or friends when seeking care [38]. Another study conducted in Malawi, a low-income country, also reported that the lack of an assistant to accompany a PWDs to health facilities as a barrier [46] Similarly, findings from a qualitative study conducted in Cameroon and India [47], reported comparable results. In our study, participants explained that they always preferred to have someone with them when seeking healthcare services to assist them physically and emotionally and not having such assistance would be a reason for not getting the care they needed. Even when participants expressed the need to have company, they felt like an extra burden on their caregivers. PWDs suffer from depression, loneliness, and believe to be a burden on their families [17, 48]. Psychological support was not something participants aimed to seek. Aligning with the Arab culture, there is still a culture of refusal in accepting psychological help among many, mainly due to stigma and misconception [49]. The study also showed that people with disabilities were unaware of the services offered. Lack of information and knowledge regarding healthcare services, referrals, and centers has been already reported elsewhere [5, 10, 47].

### COVID-19- related barriers

The changes caused by the COVID-19 pandemic further altered social and lifestyle habits and exacerbated existing barriers. With lockdown measures and physical distancing, PWDs became even more vulnerable, as they depend on services and other people to meet their needs, making them more susceptible to the virus, particularly in the Global South [18]. Unfortunately, there is no data on emergency planning for PWDs, and existing guidelines are not consistent or do not apply to the COVID-19 pandemic [50]. The COVID-19 precautionary measures are sources of stress and fear for many PWDs, especially when seeking in-person care [51].

Additionally, PWDs might fail to get the proper maintenance for their devices; also, they might fail to seek medical treatment and rehabilitation, increasing chronic, acute, and secondary health conditions [17, 51]. Even before the COVID-19 pandemic, PWDs were at higher risk of social isolation and loneliness [52]. The public health guidelines of lockdowns and physical distancing created further social exclusion among PWDs, who cannot self-isolate or be physically distant, as they rely on assistance for personal care, medication, and food delivery. The measures to mitigate the spread of the virus lead to more social isolation, segregation, creating more mental health strain, instead of protecting and supporting PWDs’ rights and needs [53].

## Strengths and limitations

This is the first study that investigated the perspective of people living with lower limb amputation, and diabetes, in accessing healthcare services during the COVID-19 pandemic in Lebanon. The study portrays a unique perspective of PWDs living in a country with protracted crises. However, some limitations should be acknowledged. The views represented in this study do not necessarily represent the entire population of PWDs. In this study no participants of Palestinian origin agreed to participate, making this segment of the population one of the most marginalized and hard to reach groups in the country. It would be important to include such a population in future inquiries, as they have different statuses compared to other more recent refugees. Moreover, the study lacks equal gender representation, as only one female participant agreed to participate (but the answers were provided mostly by her son, a male caregiver). Future studies should be conducted to represent women’s experiences and challenges in that context. The presence of some caregivers during interviews might have influenced some of their answers. The participants of the study were invited to participate by ICRC personnel, a fact that could introduce some response or social desirability bias. This also limits our understanding of the barriers of other refugees and citizens living in rural areas and not receiving any care from organizations.

## Implications for practice and future research

Based on the findings of the study, the following recommendations are addressed to humanitarian organizations offering services for PWDs:

### Reduce gender gap

Efforts should be put by communities and organizations in improving access to healthcare for women with disabilities through advocacy and education and taking into consideration gender differences when developing health interventions. There should be interventions to promote health and gender equity and empower women with disabilities to advocate for their own health. In addition, there is a need to raise awareness about the rights and the situation of women with disabilities through minimizing physical and social barriers. Healthcare workers should be trained to address women with disabilities’ needs and preferences with respect and without discrimination [31]. ICRC operations are recommended to map new ways to capture women with disabilities and to conduct future research and projects to ensure representativeness of women with disabilities. In addition, future research should identify the factors affecting the health-seeking behavior of women with disabilities and chronic diseases, to mitigate the barriers related to accessing health services.

### Reduce financial burden

There is a need for organizations to promote access to PWD by covering transportation fees and providing multipurpose cash transfers or conditional cash transfers, which have been shown to remove financial barriers, and improve access to medications and improve mental health in LMICs. In addition, conditional cash transfer has been shown to have consistently better clinical outcomes when coupled with health education in persons with diabetes [54–56]. There should also be coverage of transportation fees as it was a common barrier to accessing health services. The available literature on cash transfers addresses refugees, vulnerable host populations and/or persons with chronic diseases, however, it does not specifically address persons with disabilities and chronic diseases. Therefore, there is a need to understand the effect of cash transfers on the health clinical outcomes of persons with disabilities and chronic diseases [57].

### Reduce structural barriers

At the household levels, organizations offering rehabilitation services can better assess PWDs’ structural environment and adapt their treatment plan to accommodate the individuals’ physical surroundings. Also, organizations are called to invest more in preventive measures. Diabetic-related amputations can be avoided among people with diabetes who have received the right preventive care. Organizations can design interventions related to healthy foot care practices, which can decrease foot ulcers and eventually amputations [58].

### Reduce personal challenges

To address the barrier related to knowledge, organizations offering services for PWDs can increase their service visibility through directories and a hotline number for assistance and guidance. These directories include names and numbers of facilities and their offered services. Manuals can be distributed at hospital discharge, through health care facilities, or at the person’s doorsteps.

### Reduce cultural challenges

The needed effort is in launching campaigns that promote the societal model and “de-medicalize and de-stigmatize” disability [18]*.* Organizations and associations can promote empowerment by design and implement interventions that aim to reintegrate PWDs back into society, especially after hospital discharge. Launching virtual or in-person recreational activities, vocational training, and workshops can create opportunities for PWDs to join the workforce, be financially dependent, and alleviate the burden on caregivers.

### Reduce challenges during COVID-19

The legal frameworks regarding the right of PWDs to accessing healthcare are limited and are not being enforced. Although people living with disabilities are major stakeholders, they do not have a seat at the table to voice out their concerns and identify solutions. They are not as well-included in the preparedness and response planning leaving PWDs with no rights and outside coverage. In light of the COVID-19 pandemic, there is a need to have a tailored approach and engage PWDs in contributing and planning emergency responses [50]. Organizations should as well advocate for the proper implementation of Law 220/2000 and ratification of the UN Convention on the Rights of Persons with Disabilities and its Optional Protocol to ensure equity in service provision and accessibility [39].

## Conclusion

People living with diabetes and physical disability faced and are still facing major challenges in accessing healthcare services, including the Lebanese economic crisis and the COVID-19 pandemic coupled with various structural, cultural, and personal barriers. The uptake of healthcare services cannot depend solely on individuals’ intentions, especially when these individuals have been marginalized and isolated due to their health conditions. International and local non-governmental organizations are called to ensure a more equitable access to healthcare for people living with diabetes and physical disabilities. Urgent policies and actions are needed to address the unprecedented health, social, and economic crises that people living with disabilities are facing in Lebanon, as these crises might have detrimental long-term repercussions on their lives.

## Supplementary Information

**Additional file 1.** Interview Guide.

## Data Availability

The qualitative data generated and/or analyzed by thus study are not publicly available due to integrity of participants. De-identified transcripts of interviews may be available from the corresponding author on reasonable request.

## Abbreviations

BML: Beirut Mount Lebanon
CRPD: Convention on the Rights of Persons with Disabilities
ICRC: International Committee of the Red Cross
MoSA: Ministry of Social Affairs
NSSF: National Social Security Fund
LMIC: Low and Middle-Income Countries
PHC: Primary Health Care
PRP: Physical Rehabilitation Program
PWD: Persons with Disabilities
UNHCR: United Nations High Commissioner for Refugee
UNRWA: United Nations Relief and Works Agency
VASyR: Vulnerability Assessment for Syrian Refugees

## Footnotes

1 “Wasta” is an Arabic word that could be translated to favoritism or nepotism. It is a way of achieving objectives by using the influence of acquaintances.
