# Supplementary material for: Access to primary and secondary health care services for people living with diabetes and lower-limb amputation during the COVID-19 pandemic in Lebanon: a qualitative study
Source: BMC Health Serv Res. 2022 May 3;22:593. doi: 10.1186/s12913-022-07921-7 (PMC9063244; doi:10.1186/s12913-022-07921-7)
Supplement: Supplementary file 1 — Additional file 1. Interview Guide. [file 12913_2022_7921_MOESM1_ESM.pdf]

Additional File- Interview Guide

1. Besides being diabetic and physically disabled, do you suffer from other diabetic complications? (hearing impairment, eye damage, kidney damage, CVD..) What are they?
2. Tell me more about your diabetes. What do you do to control your blood sugar level? (Insulin, medications, proper diet…)
3. How do you usually get your medications?
4. From where do you get your medications? (Pharmacy, PHC, hospital…)
5. What kind of challenges do you face when you need to provide your daily medications?
6. And how are you affected by today's COVID-19 pandemic?
7. Do you own a glucometer or a blood pressure monitor at home? If not, where do you test yourself?
8. What about lab blood tests, where do you do them? How do you manage to get your tests done? If you cannot get tested, why cannot you do so? What are your challenges in getting tested?
9. Do you consult a nutritionist to help you with your diet? If not why so?
10. How confident do you feel about your knowledge regarding diabetes and its complications?
11. If you feel confident or not confident, what would you like to know more about it?
12. How often do you visit your physician?
13. Do you tend to miss your appointments? If yes why so? If not, what helps you keep your appointments on schedule?
14. Do you feel comfortable going to your physician's clinic today? If not why so? If yes, why so?
15. How do you describe your relationship with your physician?
16. In case you need hospitalization, how do you usually cover medical expenses?
17. Tell me more about your physical disability and ICRC rehabilitation program. How often do you need to visit the center?
18. How far is the center from your place of residency? How do you usually get to the center?
19. In case of discomfort or issues with your walking device today, do you directly consider going to the center? If not why so?

20. What about physiotherapy do you still need them? If yes how do you access this service?
21. Do you have a job? How does your job status affect you getting the health care you need?
22. In general, do you believe healthcare centers and pharmacies are easily accessible to people with disabilities? In your opinion what should be done?
23. How do you describe the treatment offered by healthcare workers in Lebanon toward people with disabilities?
24. Have you ever needed medical imaging or screening? If yes, how do you describe that experience?
25. Have you ever considered mental health support? If not, why wouldn't you consider it?
26. In case you need a dentist appointment, do you consider going today? If not, what could be a challenge?
27. If you ever need information or guidance regarding healthcare (or COVID-19 related) do you know where to go or to whom to refer to?
28. Do you feel comfortable receiving all healthcare services on your own? Or do you prefer having a companion? Why?
